# Parameters predicting postoperative pain and quality of life after hemorrhoidectomy: follow-up results from a prospective multicenter randomized trial

**DOI:** 10.1007/s00384-023-04557-9

**Published:** 2023-11-03

**Authors:** Christoph Mallmann, Mike Ralf Langenbach, Razvan-Valentin Florescu, Andreas Köhler, Jörg Barkus, Jörg-Peter Ritz, Florian Gebauer, Rolf Lefering, Lars Boenicke

**Affiliations:** 1 Department of General, Visceral and Oncological Surgery, Helios University Hospital Wuppertal, University of Witten/Herdecke, Wuppertal, Germany; 2 Ev. Krankenhaus Lippstadt, Lippstadt, Germany; 3 Helios St. Elisabeth Klinik Oberhausen, Oberhausen, Germany; 4 Helios Klinikum Duisburg, Duisburg, Germany; 5 Helios Klinikum Niederberg, Velbert, Germany; 6 Helios Klinikum Schwerin, Schwerin, Germany; 7 Institute for Research in Operative Medicine (IFOM), University of Witten/Herdecke, Witten, Germany

**Keywords:** Hemorrhoids, Hemorrhoidectomy, Quality of life, Multicenter, Predictive, Pain

## Abstract

**Purpose:**

Pain and reduced quality of life (QoL) are major subjects of interest after surgery for hemorrhoids. The aim of this study was to find predictive parameters for postoperative pain and QoL after hemorrhoidectomy.

**Methods:**

This is a follow-up analysis of data derived from a multicenter randomized controlled trial including 770 patients, which examines the usefulness of tamponade after hemorrhoidectomy. Different pre-, intra-, and postoperative parameters were correlated with pain level assessed by NRS and QoL by the EuroQuol.

**Results:**

At univariate analysis, relevant (NRS > 5/10 pts.) early pain within 48 h after surgery was associated with young age (≤ 40 years, *p* = 0.0072), use of a tamponade (*p* < 0.0001), relevant preoperative pain (*p* = 0.0017), pudendal block (*p* < 0.0001), and duration of surgery (*p* = 0.0149). At multivariate analysis, not using a pudendal block (OR 2.64), younger age (OR 1.55), use of a tamponade (OR 1.70), and relevant preoperative pain (OR 1.56) were significantly associated with relevant early postoperative pain. Relevant pain on day 7 was significantly associated only with relevant early pain (OR 3.13, *p* < 0.001). QoL overall remained at the same level. However, *n* = 229 (33%) patients presented an improvement of QoL and *n* = 245 (36%) an aggravation. Improvement was associated with a reduction of pain levels after surgery (*p* < 0.0001) and analgesia with opioids (*p* < 0.0001).

**Conclusion:**

Early relevant pain affects younger patients but can be prevented by avoiding tamponades and using a pudendal block. Relevant pain after 1 week is associated only with early pain. Relief in preexisting pain and opioids improve QoL.

**Trial registration:**

DRKS00011590 12 April 2017.

## Introduction

Hemorrhoids are common and frequently occurring diseases in the clinical setting, and higher degree hemorrhoids require surgical treatment [1].

For prolapsing hemorrhoids, excisional hemorrhoidectomy continues to be the treatment of choice with the lowest recurrence rate [2].

Despite several advantages in surgical technique and perioperative management, most patients still consider hemorrhoidectomy to be a painful procedure [3]. Therefore, although feasible in most circumstances, outpatient surgical hemorrhoid treatment is not as widely accepted as possible [4].

Postoperative pain is a common complication following hemorrhoidectomy, and its severity is influenced by the degree of surgical trauma, the type of anesthesia, any sphincter hypertonus that may be present, secondary bacterial infections, perioperative and postoperative pain management, and individual differences in pain sensitivity [5, 6].

Strong postoperative symptoms frequently prevent outpatient hemorrhoid surgery, lengthen inpatient stays in some circumstances, and occasionally result in emergency readmissions to the hospital because of unbearable discomfort [7].

Numerous studies investigated different methods of reducing postoperative pain, such as altering the surgical technique [7–10], lowering sphincter tone [11–13], taking warm sitz baths [14], giving antibiotics [15], and injecting local anesthetics into the perianal canal [7, 16].

Higher satisfaction, earlier mobilization, quicker recovery, and fewer healthcare costs can all result from adequate pain treatment [17]. Although some of the investigated interventions lead to significant improvements, the results showed a remarkable width of range in pain perception of the individual patient [3].

Accordingly, it seems to be crucial not only to develop a pathway for optimal perioperative surgical and anesthesiologic modulation but also to be able to identify those patients at risk for intensified postoperative discomfort.

Earlier, we reported the results from a multicenter, randomized clinical trial comparing the effects of placement of a tamponade dressing after hemorrhoidectomy on postoperative pain development and bleeding complications (The NoTamp study, DRKS00011590) [18]. In our extensive data set, we also noticed a strong variability in pain perception.

In the present study, we therefore reanalyzed our data to determine risk factors for the development of postoperative pain and confined health-related quality of life.

## Materials and methods

### Study design

The NoTamp study was a German multicenter randomized controlled trial (RCT) designed to compare the effects of perioperative placement of a rectal tamponade on postoperative pain development and occurrence of surgically relevant postoperative bleeding in open hemorrhoidectomy [18]. The study protocol has been described previously [19]. For further information, please refer to the registration at the German Registry of Clinical Studies (DRKS00011590) and on the study webpage (https://notamp.de/).

The trial duration for each randomized patient was 7 days. There was no blinding of participants, physicians, nurses, or outcome assessors. The study received full ethics committee approval of the University of Witten/Herdecke, Germany, and was conducted in accordance with Good Clinical Practice guidelines, with regular external monitoring.

### Cohort participants

The target population for this study included adult patients (18 years or above) suffering from symptomatic grade III or IV hemorrhoids requiring Milligan-Morgan or Parks hemorrhoidectomy [1]. Study participants were required to be fully legally competent and provide written informed consent before randomization. Patients with inflammatory anal diseases such as abscesses, fistulas, or gangrene, and pregnant women were excluded. Patients taking anticoagulatory drugs were eligible and continued taking their medication before and after surgery, in line with local standards of care. For clinical staging of hemorrhoid disease, we used the standard 4 stage Goligher’s classification [1].

The study protocol did not interfere with standard perioperative measures, and all drugs and treatments administered were recorded in case report forms (CRF).

The study recorded details such as age, sex, classification of hemorrhoidal pathology before surgery, comorbidities with impact on HD, type, and length (in minutes) of surgical procedure, duration of hospitalization (HT), type of complications developed and their time of onset, in respect to the initial procedure (Table 1). All AEs (AE) and severe AEs (SAEs) occurring in temporal relation to the clinical trial were documented.

**Table 1:** Patient and treatment parameters related to maximum pain level within 72 h after surgery. Differences were calculated using the Fisher’s exact test on the binary data, chi-squared test on nominal data, and Mann–Whitney’s *U*-test on ordinal data. Metric values are given as mean ± SD

Potential predictors for early pain	NRS 0–5 *n* = 387	NRS 6–10 *n* = 330	*p*
Age, years, mean	54.1 (14.7)	50.5 (15.0)	**0.0021**
Age < 40 y	65 (16.8%)	83 (25.2%)	**0.0072**
Sex: male	220 (56.8%)	190 (57.6%)	0.880
Weight, kg, mean	82 (18)	83 (20)	0.947
Height, m, mean	1.73 (0.10)	1.72 (0.10)	0.392
Body mass index (BMI)	27.3 (5.6)	27.7 (5.8)	0.623
Preoperative pain (median, IQR)	1 (0–3)	2 (0–4)	**0.0017**
Relevant preop. pain (> 5)	36 (9.3%)	52 (15.8%)	0.012
Goligher’s Grad IV	165 (42.6%)	164 (49.7%)	0.0606
Tamponade	170 (43.9%)	186 (56.4%)	** < 0.0009**
Anesthesia			0.589
General	348 (89.9%	289 (87.6%	
Local/spinal	16 (4.1%)	18 (5.5%)	
Others	23 (5.9%)	23 (7.0%)	
Pudendal block	71 (18.3%)	26 (7.9%)	** < 0.0001**
Milligan/Morgan	346 (89.4%)	290 (87.9%)	0.555
Duration surgery (min)	28.4 (13.9)	31.4 (15.8)	**0.0149**
Prolonged duration 40 + min	75 (19.3%)	83 (25.4%)	0.0700
Preop Hb value (mg/dl)	13.5 (2.5)	13.5 (2.4)	0.813
Comorbidity	241 (62.3%)	185 (56.1%)	0.0938
DVT prophylaxis	262 (67.7%)	210 (63.6%)	0.267

Significant differences (*p*<0.05) are given in bold

In the present spin-off study, we questioned the already existing data set for variables predicting the development of relevant postoperative pain.

Pain intensity was determined at various time points using a Numeric Rating Scale (NRS, ranging from 0 to 10), namely, at 6, 12, 24, and 48 h, as well as on postoperative days 3 and 7. Relevant postoperative pain was defined as NRS rating higher than 5 points. At this pain level, most patients require intense pain medication (e.g., opioids) [20, 21].

The primary endpoint was to identify factors associated with early relevant pain, which was defined as maximum NRS measurement from five postoperative time points up to day 3.

The secondary endpoint was to identify predictors for relevant pain at 1 week postoperatively (day 7).

Finally, the change in the patients’ quality of life from pre- to postoperative was examined by utilization of the EuroQoL Group (Rotterdam, The Netherlands) index (EQ-5D™) on generic health-related quality of life at screening and on day 7 [22].

### Statistics

Statistical analysis was performed using IBM SPSS Statistics for Windows, Version 28. Armonk, NY: IBM Corp. All categorical variables were presented as counts (with percentages) and were analyzed with chi-squared or Fisher’s exact test. Continuous data were summarized as mean with standard deviation (SD), or median with interquartile range (IQR), as appropriate, and were compared with Mann-Whiney *U*-Test. In case of three groups (quality of life), chi-squared test and Kruskal–Wallis were used, respectively. Multivariate logistic regression analysis has been performed with relevant pain as dependent variable, and potential predictors available until the end of surgery as independent variables (age, sex, BMI, type of anesthesia, prior illness, use of a tamponade, pudendus block, relevant pre-operative pain, type of surgery, and grading). For prediction of relevant late pain, early (days 1–3) pain was also included. Results are presented as odds ratios (OR) with 95% confidence intervals (CI_95_) and respective *p* value.

## Results

### Study cohort

The main data set has been published previously (NoTamp) [18]. Between May 2017 and November 2020, 950 patients were screened for eligibility; of these, 725 patients were enrolled at 14 participating hospitals. Further, 8 patients were excluded from this analysis due to missing relevant data for pre- or postoperative pain. Therefore, 717 cases were enrolled for the statistical exploration. Table 1 shows patients’ history and other clinical parameters of the study cohort.

### Primary endpoint: relevant early pain

**Fig. 1:**
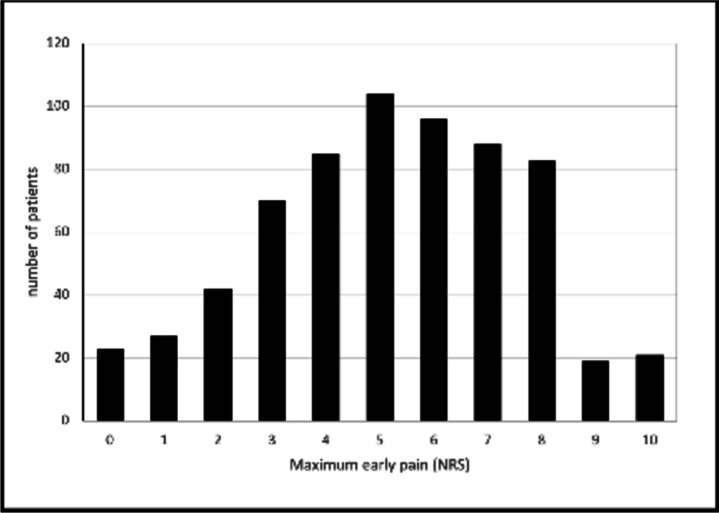
Distribution of the maximum postoperative pain levels measured by the NRS within the study cohort in the first 3 days after surgery

Figure 1 shows the prevalence of the maximum pain level observed until day 3. The median (IQR) pain was 5 (3–7), with an average value of 5.1 points. 330 out of 717 patients (46%) experienced relevant pain with NRS levels 6–10. Only 38.6% of the patients did not reach 5 on the NRS within the study period.

### Predictors for early relevant pain

We checked possible predictors in both subgroups with and without relevant postoperative pain until day 3. Univariate analysis revealed that younger age, preoperative pain level, long duration of surgery, and usage of a tamponade were associated with relevant pain after surgery (Table 1). Patients with relevant pain more often received opioids in the postoperative phase, as expected (41% versus 30%, *p* = 0.0062).

In the multivariate analysis, pudendal block was the strongest parameter. Without a pudendal block,the risk for relevant postoperative pain doubles (OR 2.64, CI_95_ 1.61–4.31, *p* = 0.0001). Use of a tamponade (OR 1.70, CI_95_ 1.25–2.32, *p* = 0.0007), young age up to 40 years (OR 1.55, CI_95_ 1.04–2.31, *p* = 0.0334), relevant preoperative pain (OR 1.56, CI_95_ 0.96–2.52, *p* = 0.0700) also increase the likelihood of relevant early postoperative pain. All other predictors had minor effects with *p* > 0.10.

### Secondary endpoint: relevant pain after 1 week

**Fig. 2:**
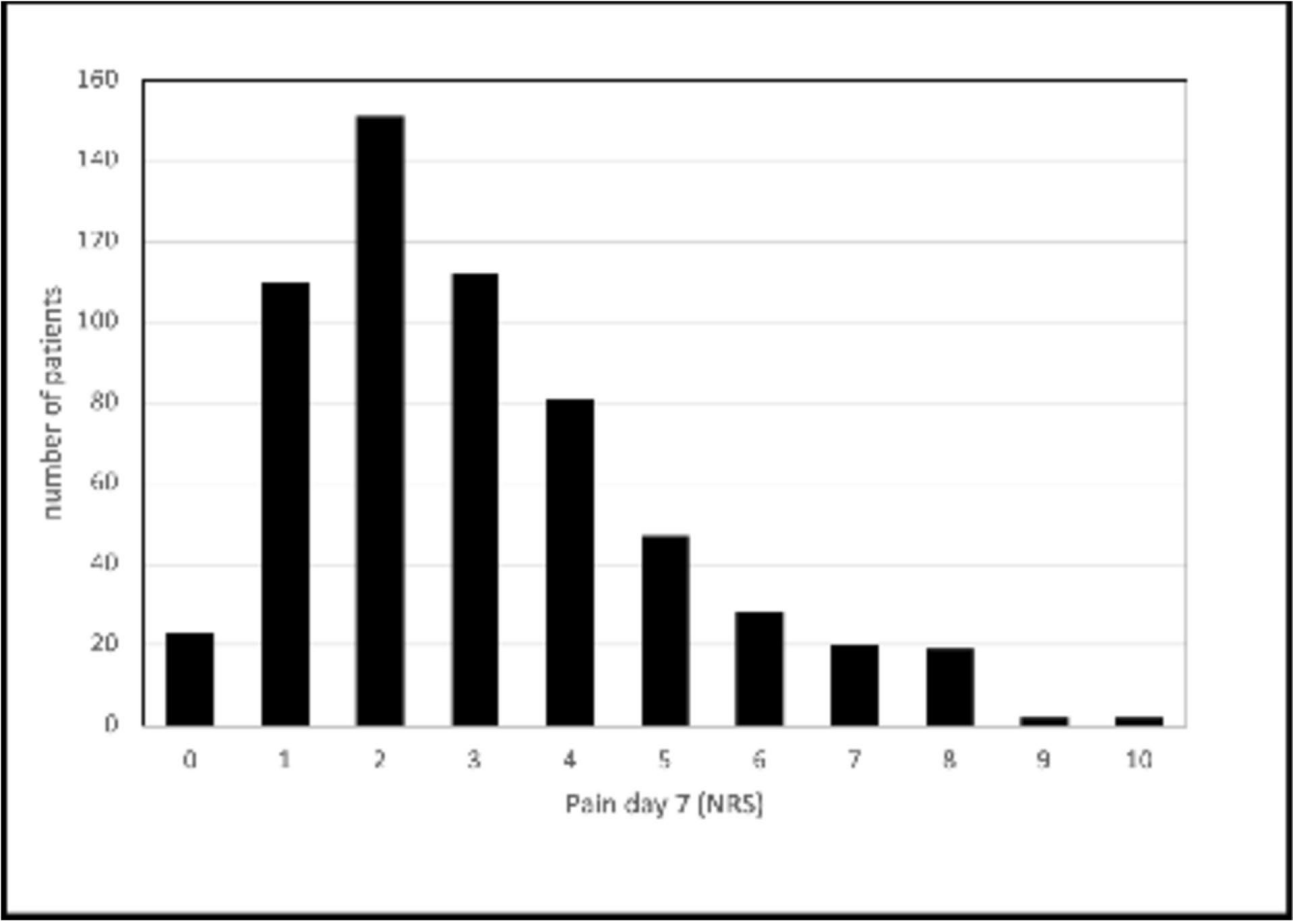
Distribution of the postoperative pain levels measured by the NRS within the study cohort 7 days after surgery

Secondary endpoint was relevant delayed pain as shown by the NRS > 5 on day 7. For this time point, data of 688 of the 717 in patients (96%) were available. The mean and median (IQR) NRS values at that time were 2.7 and 2 (1–4), respectively. Seventy-four patients (10.8%) complained about relevant pain (NRS 6–10) on day 7 (Fig. 2).

### Predictors for relevant delayed pain

Relevant early pain was the strongest predictor for relevant pain after 7 days (OR 3.13, CI95 1.81–5.41, *p* < 0.0001). All other potential predictors had an OR < 2.0 and a *p* > 0.10. Relevant preoperative pain had no association with pain on day 7 (Table 2).

**Table 2:** Patient and treatment parameters related to maximum pain level 7 days after surgery. Differences were calculated using the Fisher’s exact test on the binary data, chi-squared test on nominal data, and Mann–Whitney’s *U*-test on ordinal data. Metric values are given as mean ± SD

Potential predictors for delayed pain	NRS 0–5 *n* = 614	NRS 6–10 *N* = 74	*p*
Age, years, mean (SD)	52.4 (15.0)	53.4 (14.3)	0.808
Age < 40 y	128 (20.8%)	12 (16.2%)	0.445
Sex: male	353 (57.5%)	42 (56.8%)	0.902
Body mass index (BMI)	27.5 (5.8)	28.5 (5.4)	0.0698
Preoperative pain (median, IQR)	1 (0–3)	2 (0–4)	0.13
Relevant preop. pain (> 5)	75 (12.2%)	10 (13.5%)	0.710
Goligher’s Grad IV	290 (47.2%)	29 (39.2%)	0.218
Tamponade	306 (49.8%)	33 (44.6%)	0.460
Pudendal block	88 (14.3%)	8 (10.8%)	0.481
Duration surgery (min)	29.2 (14.3)	34.2 (17.8)	**0.0310**
Prolonged duration 40 + min	125 (20.6%)	23 (31.1%)	0.0510
Relevant postop pain (> 5)	269 (43.8%)	52 (70.3%)	** < 0.0001**
Further outcomes			
Postop bleeding	15 (2.4%)	2 (2.7%)	0.703
Adverse events	41 (6.7%)	7 (9.5%)	0.339

Significant differences (*p*<0.05) are given in bold

### Third endpoint: quality of life (QoL)

Quality of life was assessed preoperatively and with the follow-up on day 7. In the preoperative setting, the indices of 713 of 717 patients were assessed, postoperatively of 690 cases. For this evaluation, we only used pre- and postoperative complete data sets of 686 cases.

The mean/median preoperative quality of life index overall was 82.3/92.6 (IQR 79.5–92.6). One week after surgery, the mean/median values were rather similar: 83.6/92.6 (IQR 79.5–92.6).

Although this seems to show an unaltered quality of life throughout the perioperative setting, the in-detail comparison of the intra-individual changes draws a different picture.

**Fig. 3:**
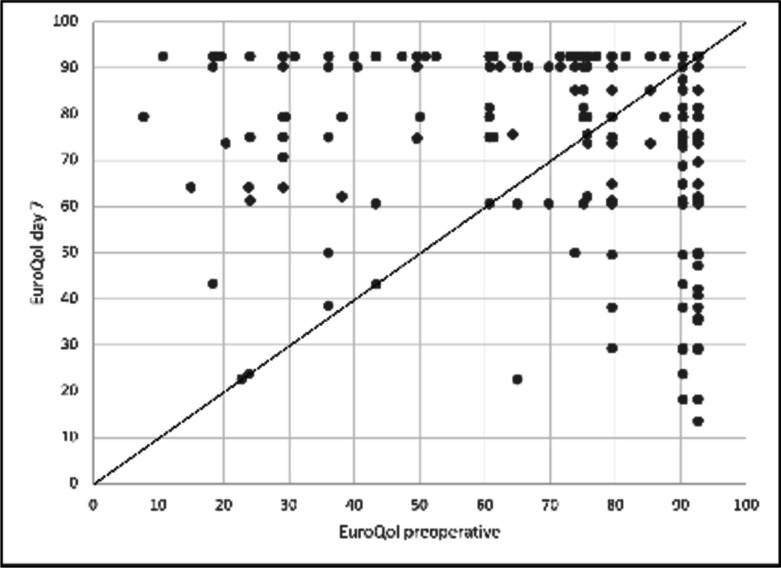
Combined distribution of pre- and postoperative values of the EuroQoL. Every dot represents one or more patients. Patients on the diagonal (31%) show an unchanged quality of life

The dot plot in Fig. 3 shows the combined distribution of pre- and postoperative values. Every dot in the figure represents one or more patients. Patients on the diagonal line show an unchanged quality of life (*n* = 212, 30.9%). Patients above the diagonal line (*n* = 229, 33.4%) show an increased QoL after surgery while the dots below the diagonal line (*n* = 245, 35,7%) present those with a worsened QoL. As the number of patients with improved and worsened QoL were nearly identical, the values observed before and after surgery were rather similar. Table 3 shows different parameters in the individual groups.

**Table 3:** Patient and treatment parameters subdivided in patients with lower, unchanged, or higher QoL as shown by the EuroQol after surgery. Differences were calculated using the Fisher’s exact test on the binary data, chi-squared test on nominal data, and Mann–Whitney’s *U*-test on ordinal data. Metric values are given as mean ± SD

	QoL lower *N* = 245	QoL unchanged *N* = 212	QoL higher *N* = 229	*p*
Age, years, mean (s.d.)	51.2 (14.4)	54.0 (14.8)	52.3 (15.0)	0.189
Sex: male	145 (60%)	122 (58%)	123 (54%)	0.426
QoL Index pre (SD)	90.4 (5.7)	91.1 (7.7)	65.6 (21.6)	< 0.0001
QoL Index post (SD)	72.2 (17.8)	91.1 (7.7)	88.9 (8.3)	< 0.0001
Pain pre (median IQR)	0 (0–2)	0 (0–2)	4 (2–6)	** < 0.0001**
Max pain postop. (median IQR)	5 (3–7)	5 (4–7)	5 (4–7)	0.503
Pain on day 7 (median IRQ)	2 (1–4)	2 (1–4)	2 (1–4)	0.416
More pain on day 7	149 (63%)	132 (64%)	65 (30%)	** < 0.0001**
Relevant preop. pain	10 (4%)	6 (3%)	66 (29%)	** < 0.0001**
Relevant postop. pain	118 (48%)	104 (49%)	96 (42%)	0.300
Relevant pain on day 7	34 (14%)	15 (7%)	25 (11%)	0.0742
Tamponade	130 (53%)	95 (45%)	114 (50%)	0.258
Duration surgery (min) (SD)	30.2 (13.6)	27.6 (13.3)	31.3 (15.2)	0.0832
Opioid treatment	76 (31%)	57 (27%)	110 (48%)	** < 0.0001**
Adverse events	15 (6%)	15 (7%)	18 (8%)	0.745

Significant differences (*p*<0.05) are given in bold

As shown in Table 3, the group of patients who described an increase in quality of life after surgery predominantly had a higher preoperative pain score (median NRS 4) as compared to the group with a worsening of QoL (median NRS 0, *p* < 0.0001). More pain than preoperatively was observed in 63% and 64% of patients with a worse or equal QoL, respectively, while those with a better QoL only reported more pain in 30% (*p* < 0.0001). Utilization of opioids within the first 48 h as part of the postoperative pain management was also associated with a gain in QoL (*p* < 0.0001).

Pain level early postoperative or on day 7, surprisingly, did not lead to a decline in QoL. Overall, in our data set, we were not able to point out significant predictive measures for a quality-of-life drop postoperatively. This group of patients had only marginal higher early postoperative pain (mean NRS 2.9 ± SD 2.2), and relevant postoperative pain on day 7 was prevalent in only 14% of cases. Adverse events or bleeding did not occur more often compared to the other groups.

## Discussion

For prolapsing hemorrhoids, excisional hemorrhoidectomy continues to be the treatment of choice with the lowest recurrence rate [2].

However, most patients consider hemorrhoidectomy to be a painful procedure and are afraid of the operation [3]. Therefore, it seems to be crucial not only to develop a pathway for optimal perioperative surgical and anesthesiologic modulation but also to be able to identify those patients at risk for intensified postoperative discomfort.

In the present study, we reexamined our previously published data of the NoTamp study (DRKS00011590) on the effect of postoperative placement of a tamponade dressing in open hemorrhoidectomy [18] with a focus on factors leading to relevant postoperative pain and change in quality of life.

To our knowledge, this is so far the prospective study with the largest study cohort to analyze possible predictors of relevant early postoperative pain, delayed relevant pain, and changes in quality of life after open hemorrhoidectomy.

The main finding was that the pudendal block as part of the anesthesiologic concept reduced the risk of relevant postoperative pain on day 3 by half. Other factors that increased the risk of relevant postoperative pain were young age, relevant preoperative pain, and tamponade use. At univariate analysis, the duration of surgery was significantly associated with the occurrence of relevant early and delayed pain on day 7. Postoperative complications did not have a significant effect on postoperative pain. The study also showed that patients who reported a higher preoperative pain score developed a relief in pain and an increase in quality of life 7 days after surgery. On the other hand, there were no clear predictors for a quality-of-life drop after surgery. Especially relevant pain on days 3 and 7 or postoperative complications did not show a significant correlation with QoL in our study group.

Pain is the most common postoperative complication of classic hemorrhoidectomy, occurring in most patients [23]. Our findings about the reported pain level on days 1 to 3 is in accordance with other studies on this matter [24]. The correlation between early postoperative, augmented pain, and delayed elevated pain levels is not only comprehensible but has been also reproduced in several studies [25, 26].

In our study, the use of a pudendal block reduced the risk of relevant postoperative pain on day 3 by half. This is consistent with some [27], but not all studies [28].

In a prospective, single-blinded randomized controlled trial, comparing local anesthesia with or without pudendal block, Steen et al. demonstrated no differences in postoperative pain between groups at 4 h, 8 h, 12 h, or 24 h [27]. Delayed pain was not documented. As previous studies did not use additional local anesthesia, the authors argued that application of local anesthesia in the wound bed alleviates the further benefits of a pudendal block on the overall symptom control. Also, a nerve stimulator was not used for determining block placement; therefore, it is possible that in this setting, pudendal nerve block was not actually achieved. Nonetheless, pudendal block with or without general anesthesia is still recommended for all patients undergoing hemorrhoidal surgery [28].

Younger age is known to be a risk factor for the experience of increased postoperative pain intensity, as well as the presence of preoperative pain [25].

The impact of a postoperative tamponade dressing on postoperative pain has not been previously described and let to the underlying study.

To our knowledge, the impact of a prolonged hemorrhoidectomy procedure on postoperative pain has not been demonstrated. The extended duration of surgery could be due to a more extensive resection, which naturally leads to more early and delayed pain. However, in the present study, this parameter showed only significance at univariate analysis.

In recent years, variations in surgical technique for hemorrhoidal dissection have been invented with the goal of minimizing pain without increasing the risk of postoperative adverse events such as bleeding and the risk of recurrence [7–10]. Postoperative pain in these various surgical procedures has been explicitly examined in several clinical trials, reviews, and meta-analyses [29].

Early pain relief measures, anal sphincter relaxation, wound healing, remission of edema and congestion, and breaking the vicious cycle of “pain-sphincter spasm-pain aggravation” are crucial factors in postoperative pain management [25]. However, most studies fail to report data on delayed pain after open hemorrhoidectomy. Overall, data from a systematic meta-analysis comparing the influence of conventional open hemorrhoidectomy, considered the reference technique, against other surgical procedures such as closed hemorrhoidectomy, open hemorrhoidectomy using bipolar or ultrasonic sealant, hemorrhoidopexy, or HAL-RAR (Haemorrhoidal Artery Ligation and Recto Anal Repair) on post-operative pain in an out-patient setting is in line with the reported delayed pain level of our study cohort [3, 29].

This study has a few limitations. It is likely that some important parameters that contribute to the perception of pain and quality of life have not been examined, such as personality profile, education, or social status. Also, psychosocial factors as common risk factors for postoperative pain have not been examined.

In addition, a longer follow-up period would be desirable.

Taken together, this study identified predictors for the development of relevant early postoperative pain and delayed pain and may help identifying those patients at risk. Especially for these patients, systematic standardized therapeutic pathways incorporating current scientific findings could lead to a higher acceptance of smaller colorectal surgery as an outpatient procedure.

The fear of worsening pre-existing pain and more reduced quality of life after a hemorrhoidectomy seems unfounded; such patients clearly benefit from the operation.

As consequence, adequate pain management including opioids and avoiding unnecessary measures such as tamponades are important.

## Data Availability

Data will be made available at the website of the NoTamp study. Anonymized raw data are available upon request from the corresponding author or the trial statistician.
